# Hydrologic cost-effectiveness ratio favors switchgrass production on marginal croplands over existing grasslands

**DOI:** 10.1371/journal.pone.0181924

**Published:** 2017-08-08

**Authors:** Yohannes Tadesse Yimam, Tyson E. Ochsner, Garey A. Fox

**Affiliations:** 1 Department of Soil and Crop Sciences, Texas A&M University, College Station, Texas, United States of America; 2 Department of Plant and Soil Sciences, Oklahoma State University, Stillwater, Oklahoma, United States of America; 3 Biosystems and Agricultural Engineering, Oklahoma State University, Stillwater, Oklahoma, United States of America; Universitat fur Bodenkultur Wien, AUSTRIA

## Abstract

Switchgrass (*Panicum virgatum L*.) has attracted attention as a promising second generation biofuel feedstock. Both existing grasslands and marginal croplands have been suggested as targets for conversion to switchgrass, but the resulting production potentials and hydrologic impacts are not clear. The objectives of this study were to model switchgrass production on existing grasslands (scenario-I) and on marginal croplands that have severe to very severe limitations for crop production (scenario-II) and to evaluate the effects on evapotranspiration (ET) and streamflow. The Soil and Water Assessment Tool (SWAT) was applied to the 1063 km^2^ Skeleton Creek watershed in north-central Oklahoma, a watershed dominated by grasslands (35%) and winter wheat cropland (47%). The simulated average annual yield (2002–2011) for rainfed Alamo switchgrass for both scenarios was 12 Mg ha^-1^. Yield varied spatially under scenario-I from 6.1 to 15.3 Mg ha^-1^, while under scenario-II the range was from 8.2 to 13.8 Mg ha^-1^. Comparison of average annual ET and streamflow between the baseline simulation (existing land use) and scenario-I showed that scenario-I had 5.6% (37 mm) higher average annual ET and 27.7% lower streamflow, representing a 40.7 million m^3^ yr^-1^ streamflow reduction. Compared to the baseline, scenario-II had only 0.5% higher ET and 3.2% lower streamflow, but some monthly impacts were larger. In this watershed, the water yield reduction per ton of biomass production (i.e. hydrologic cost-effectiveness ratio) was more than 5X greater under scenario-I than under scenario-II. These results suggest that, from a hydrologic perspective, it may be preferable to convert marginal cropland to switchgrass production rather than converting existing grasslands.

## Introduction

The US has a goal of producing 36 billion gallons of biofuels annually by 2022 [1], primarily as ethanol. Thus far, production of ethanol in the US has been predominantly from corn grain, but future increases in biofuel production are expected to come mostly from cellulosic feedstocks [2]. Switchgrass (*Panicum virgatum*) is considered by some to be a promising cellulosic feedstock crop for much of the US, including the Southern Great Plains (SGP), the focus area for our study [3, 4, 5]. Switchgrass is a warm season C4 perennial grass native to Central and North America. High biomass production, relatively low management requirements, adaptability to poor soils, and drought resistance are some of the reasons switchgrass has been identified as a potential bioenergy crop [6].

Large scale production of bioenergy crops will require alterations in land use and land cover, which may have significant hydrologic effects [7]. Decisions about which energy crops to plant, where to grow them, and how to manage them will be important in determining effects on water resources [8]. For example, Schilling et al [9] predicted a 9.5% increase in evapotranspiration (ET) and a 28% reduction in streamflow upon converting 100% of croplands (about 76% of the watershed) to switchgrass production in the Raccoon River watershed in Iowa, USA. In the Iowa River basin, Wu and Liu [10] simulated an increase in streamflow by converting corn producing lands to switchgrass production; they also predicted a reduction in streamflow by changing grasslands to switchgrass production. By coupling land surface model with a weather forecasting model, Khanal et al [11] predicted a reduction in streamflow volume by 20% in Kansas and Oklahoma, USA as a result of increased in ET for biofuel crop production scenarios. A recent study in part of the middle North Canadian River basin in Oklahoma projected an increase in ET by 3.4 to 32% during spring and 1.5 to 18.9% during summer when both winter wheat producing areas and grasslands were converted to switchgrass production with impacts varying depending on the amount of fertilizer inputs and total area of conversions [12]. These increases in ET were predicted to result in a reduction in streamflow by 5.6 to 20.6% during spring and 6.4 to 31.2% during the summer. Similarly, in the Skeleton Creek watershed, Goldstein and Tarhule [13] predicted an increase in ET and a decrease in runoff during the spring and summer following conversion of 89% of the watershed (currently under grassland, winter wheat, and rye) to switchgrass production.

Together, these studies show that converting >80% of the land in a watershed to switchgrass production can substantially alter streamflow, with streamflow being reduced in most cases. These streamflow reductions are generally undesirable hydrological impacts which could threaten aquatic ecosystems and downstream water use. Terrestrial ecosystems would also likely experience negative impacts stemming from the loss of biodiversity if such large portions of watersheds were converted to a single plant species. There are economic disincentives to these near-total conversion scenarios, as well. Under these scenarios, much of the land newly devoted to biofuel feedstock production would come from productive farmland which was formerly devoted to food production. In response to this concern, some researchers have suggested planting bioenergy crops only on marginal lands in order to reduce competition with food crops (e.g. [14; 15]). Targeting only marginal lands would result in lower percentages of watersheds being converted to switchgrass, but, the hydrologic impacts of these less extreme land conversion scenarios are currently uncertain.

Although there have been several hydrologic modeling studies on land use conversion to switchgrass production, few have considered conversion of only marginal lands (e.g. [16, 17]). Currently, there is no widely accepted definition of marginal lands that would let us identify them on a map. This complicates regional scale studies of bioenergy production and its relation with environmental variables. Previous researchers have used a variety of definitions for marginal lands including: lands that are susceptible to degradation and low inherent productivity, hence high risk for crop production [18]; lands that are less suitable for crop production due to inherent soil or climatic limitations or lands that are venerable to environmental risks [15, 19]; abandoned agricultural lands and lands reserved for conservation, buffer strips along water bodies and roadway, and contaminated lands [20]; lands with a high runoff per unit cotton yield [17]; and lands having severe to very severe limitations for production of crops common to the area [21].

In this study, the Natural Resources Conservation Service (USDA-NRCS) land capability classification (LCC) system was used to define marginal cropland. The LCC system classifies lands based on their suitability for cultivation of crops common to the region or for pasture, range, and forest or wildlife habitat. The system has eight classes, ranging from class I, defined as land with only slight limitations that restrict crop production, to class VIII, which is defined as land that is only suitable for recreation, wildlife, water supply, or aesthetic purposes [22]. In this study we defined marginal cropland as land which has severe (class III) to very severe (class IV) limitations for crop production. The limitations can be either one or a mix of the four limitations considered for the classification, which are: susceptibility to erosion, excess water, soil limitations within the root zone, and climate [22]. A similar approach was used by Graham [21], who considered LCC class III and IV to estimate the potential land base for bioenergy crop production in the conterminous United States. Thomas et al. [23] also used LCC class III and IV as marginal soils in their modeling of water quality impacts of growing selected bioenergy crops. Likewise, Gelfand et al. [24] considered LCCs V-VII with slope gradients of < 20% as marginal lands in their assessment of the potential of ten Midwestern US states for feedstock production.

Existing grasslands may also be suitable locations for cellulosic feedstock production [18, 25]. One disadvantage of deriving bioenergy from grassland is the displacement of these lands from their current role of producing forage for grazing animals [26]. Nevertheless, farmers are more willing to replace grasslands than croplands with switchgrass [27]. Thus, existing grasslands and marginal croplands have both been identified as potential land areas for conversion to switchgrass production, but the extent to which the hydrologic impacts of conversion differ between grasslands and marginal croplands is unknown. Studies of the impacts of bioenergy production on water resources for grassland conversion versus marginal cropland conversion have not been reported for the SGP. There is a need to estimate the bioenergy feedstock production potential of these land areas and the corresponding effects on ET and streamflow, which in turn impacts aquatic ecosystems and water use downstream. In this study, the Soil and Water Assessment Tool (SWAT) [28] was applied to the Skeleton Creek watershed in north central Oklahoma 1) to estimate the rainfed switchgrass production potential on grasslands and marginal croplands and 2) to evaluate the hydrological impacts of converting grasslands versus marginal croplands to switchgrass production.

## Materials and methods

### Study area

**Fig 1:**
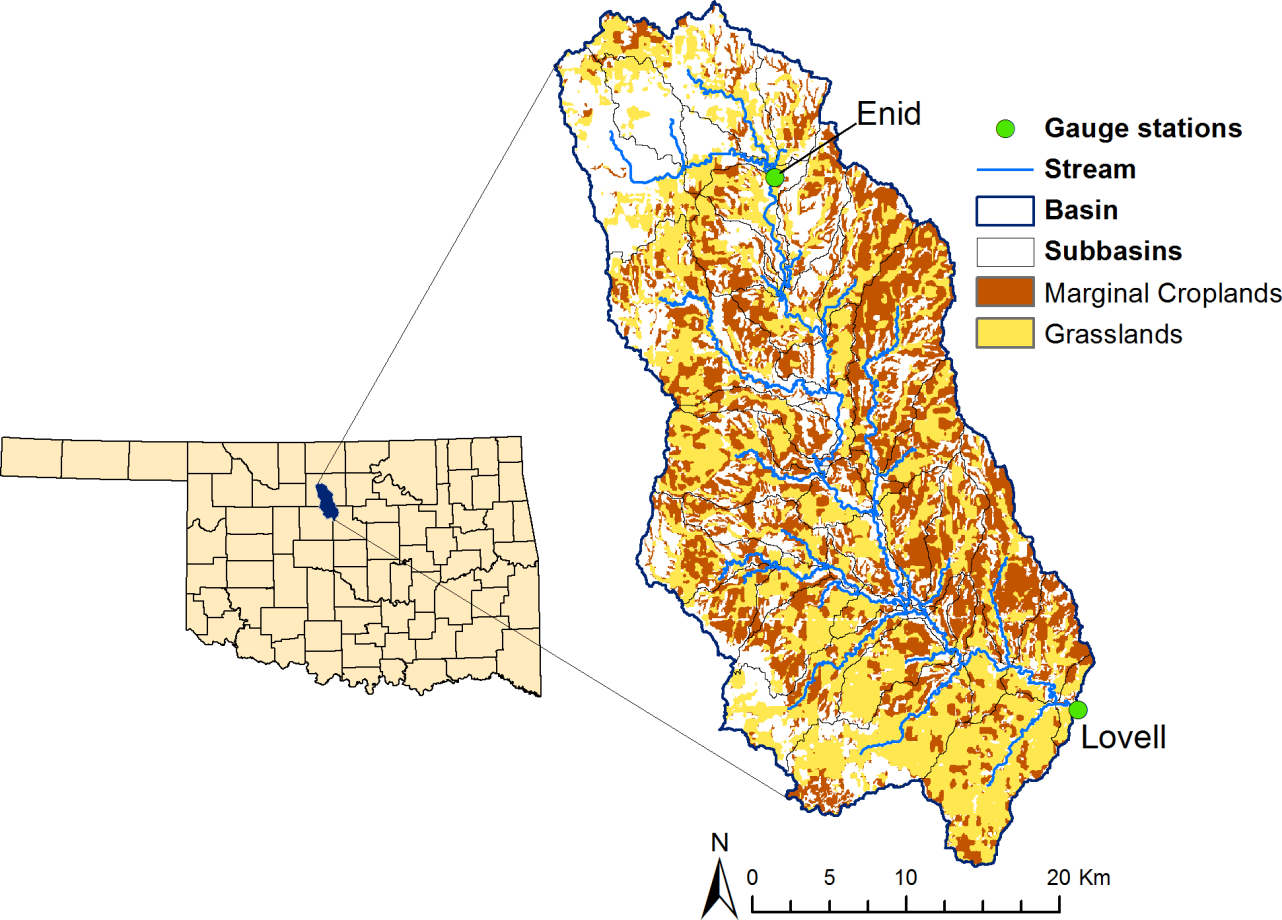
Location of Skeleton Creek watershed in north central Oklahoma, USA and distribution of grasslands and marginal croplands in the watershed.

**Fig 2:**
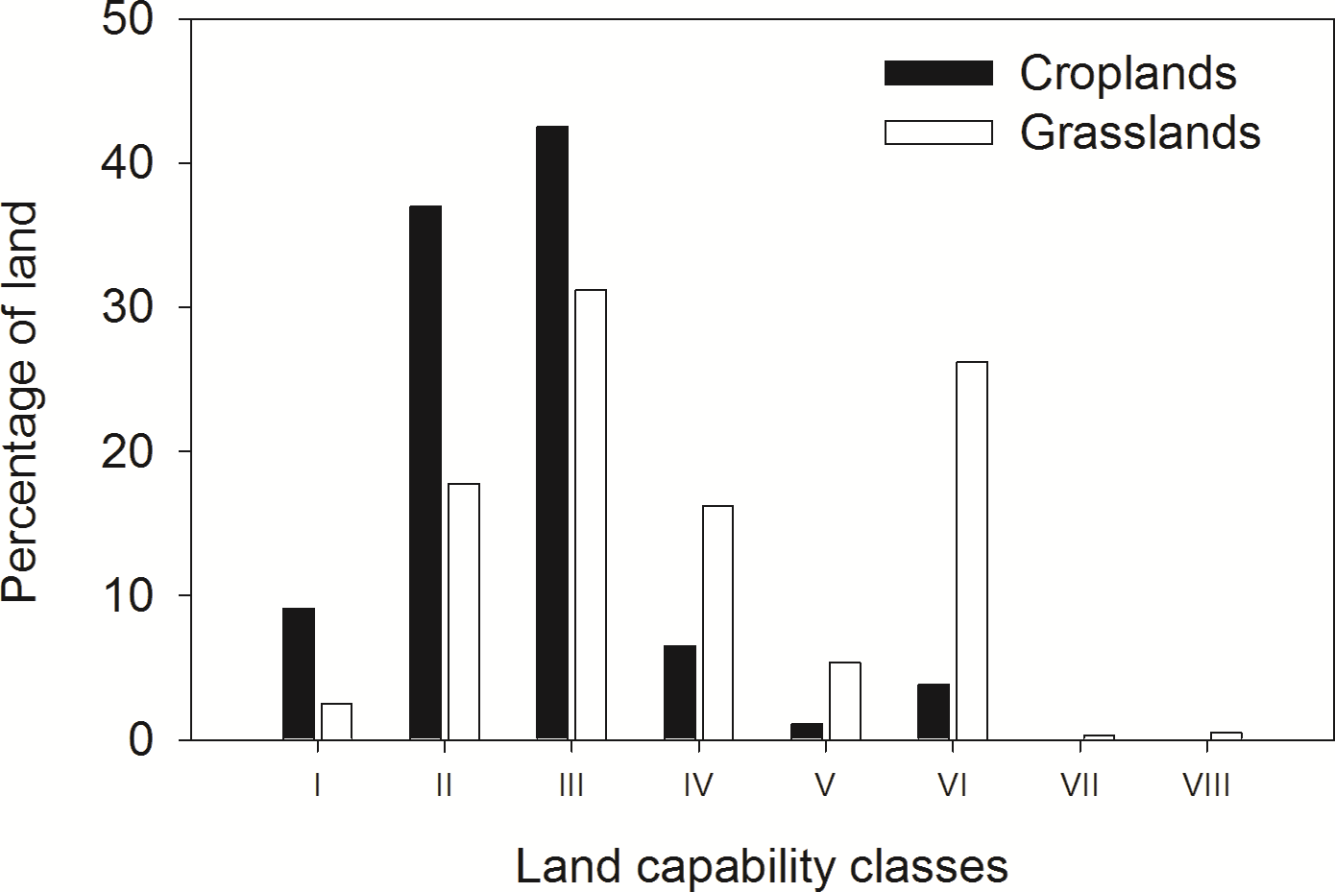
Percentages of croplands and grasslands in NRCS land capability classes I to VIII.

The Skeleton Creek watershed covers a total surface area of 1063 km^2^ and lies within three counties (Garfield, Kingfisher, and Logan) located in north-central Oklahoma (Fig 1). The watershed was delineated using the USGS streamflow station at Lovell as the outlet for the watershed. Inside the watershed, there is an additional USGS streamflow gauge station at Enid draining 16% of the total area of the watershed. More than 80% of the developed area in the Skeleton Creek watershed is located above the Enid gauge station. The majority of the watershed has fine surface soil texture, and the soil profile is grouped under taxonomic orders Mollisol and Alfisol. The elevation of the watershed ranges between 280 and 415 m above mean sea level. The watershed is relatively flat with a mean slope of 2.0%. The mean slope of the existing croplands in the watershed is 1.5%; while the grasslands have a mean slope of 2.8%. Winter wheat (Triticum aestivum L.), grassland herbaceous, and areas under different level of urban development together comprise 94.4% of the total watershed area, representing 52.1%, 35.4% and 6.8%, respectively. In the watershed, 596 km^2^ (56.1% of the total area) are currently under cultivation. About 48.4% of the cultivated land is marginal cropland in capability classes III and IV, and most of that land is used for the production of winter wheat (Fig 2). About 80% of the grasslands are in land capability class III or higher and are not well suited for crop production. The land cover in the watershed is representative of other watersheds in the SGP where winter wheat and grasslands are predominant [13].

### The SWAT model

The Soil and Water Assessment Tool (SWAT) was applied in the Skeleton Creek watershed to estimate the switchgrass production potential of grasslands and marginal croplands and to compare the hydrologic impacts of converting these two distinct land areas to switchgrass. SWAT is a physically based, semi-distributed, continuous watershed model. The model was designed to predict the effect of management decisions on water, sediment, nutrient, and pesticide yields in watersheds with varying soils, land use, and management practices [28]. The model simulates the hydrological cycle, plant growth, nutrient cycling, and pesticide and sediment transport under different management practices. SWAT has been used around the world for a wide variety of applications including simulation of land-use change and climate change impacts in various watersheds [29]. SWAT has also been used to study the effect of bioenergy crop production on water quantity and quality (e.g. [9, 30, 31]) and to simulate watershed scale bioenergy yield for economic analysis [32]. SWAT was chosen for this study because of the model’s ability to simulate spatially-explicit yield and water use for switchgrass, existing grasslands, and existing cropland in the watershed, along with simulation of streamflow and other water balance components.

A watershed in SWAT is divided into multiple sub-watersheds or sub-basins which are further divided in to a number of Hydrological Response Units (HRUs). Each HRU is made up of homogeneous land use, management, and soil characteristics. For each HRU, SWAT simulates the hydrological cycle based on the water balance of the soil profile. The water balance equation is the primary driver of the model. The model uses the Erosion Productivity Impact Calculator (EPIC) modeling approach to simulate crop growth [33]. As in EPIC, the development stage of a crop is defined in terms of daily accumulated heat units. For each day of simulation, plant growth is calculated from the daily intercepted photosynthetically active radiation and plant species specific radiation use efficiency. Harvest index is used to calculate harvested aboveground biomass from the total biomass. Detailed description of the model structure can be found in the SWAT Theoretical Manual [28].

### Input data and sources

As a physically based model, SWAT requires a substantial amount of data for model parametrization. Major input datasets for the model include climate, topography, soil, land use/ land cover, and farm management practices. The geospatial data used in this research include a digital elevation model (DEM), land cover data, and soil data. The 30-m spatial resolution DEM from the National Elevation Dataset (NED) was used to define the topography [34]. The DEM is used to calculate sub-basin parameters such as slope, aspect, and slope length, and to define the stream networks. To define the location of the stream networks in the model, the streamflow network shapefile [35] was superimposed onto the DEM. A total of 44 sub-watersheds were delineated using a minimum drainage area threshold of 1500 ha. The 1500 ha threshold was set to capture major tributaries found in the watershed. The high resolution (1:24,000 scales) Soil Survey Geographic Database (SSURGO) was used to describe the distribution and properties of the soil for the Skeleton Creek watershed [36]. Information about land cover was obtained from two different datasets to better capture both agricultural and non-agricultural land cover information. These were the 2006 National Land Cover Dataset (NLCD) [37] and the 2007 Cropland Data Layer (CDL) [38]. Since the CDL has detailed information mainly on cultivated lands, it was merged with the NLCD to determine the type of crops grown on the “cultivated crops” class of the NLCD as proposed by Srinivasan et al [39]. The NLCD was used for the non-cultivated areas.

In the SSURGO database, the Land Capability Classification (LCC) for irrigated and non-irrigated conditions are included for each soil map-unit showing the suitability of the land for the cultivation of crops common to the region. A map of non-irrigated LCC was extracted from the SSURGO database for each soil map-unit found within the watershed. By overlying this map with the land cover map it was possible to identify class III and IV lands which were being used for crop production. Once the soil and land use maps were prepared, the HRUs were created by overlaying the land use, soil, and slope maps. Since the watershed is relatively flat with a mean slope of 2.0%, a single slope class was used. A zero land use and soil type percent threshold was considered when creating the HRUs in order to be able to locate the HRUs geographically. The study area was divided into 5021 HRUs with a mean area of about 22 ha.

Daily values of precipitation, maximum and minimum air temperature, solar radiation, relative humidity, and wind speed were collected from Oklahoma Mesonet weather stations that are found inside and close to the watershed [40]. For sensitivity analysis, calibration, and validation, daily streamflow data from two gauge stations (Enid and Lovell, Fig 1) were obtained from USGS water information system (http://waterdata.usgs.gov/nwis/sw). Thirteen years (from 01/01/1999 to 12/31/2011) of weather and flow data were considered. The first three years were used for model “warm up” followed by six years for calibration and the final four years for validation.

### Plant growth parameters and management practices

The default parameters for Alamo switchgrass in the SWAT crop growth database were used with some modifications based on recent literature. To simulate a mature switchgrass stand, the initial LAI was changed from 0 to 0.5 m^2^ m^-2^ and the initial biomass was changed from 0 to 500 kg ha^-1^ [30]. The maximum LAI was allowed to reach up to 6 m^2^ m^-2^ and the roots were also allowed to grow up to 2.2 m. In addition, the radiation use efficiency was changed from 47 to 43 (kg ha^-1^)/ (MJ m^-2^), which is a more representative value for the study region, according to Kiniry et al. [41]. Winter wheat crop growth parameters were adjusted to match average grain yield in the region (~ 2 Mg ha^-1^; [42]). Existing grasslands in the watershed were simulated as range grasses in the model. The dominant grasses in the watershed include big bluestem (*Andropogon gerardi*), little bluestem (*Schizachyrium scoparium*), sideoats grama (B*outeloua curtipendula*), indiangrass (*Sorghastrum nutans*), and switchgrass (*Pancium virgatum*) [43]. We simulated the grasslands with the default SWAT crop growth parameters for ‘little bluestem’, since little bluestem is able to grow in the majority of the soils found in the watershed [43, 44, 45]. Some of the growth parameters of little bluestem include a radiation use efficiency of 34 (kg ha^-1^) / (MJ m^-2^), maximum LAI of 2.5, an optimal temperature of 25°C, and a base temperature of 12°C.

The management operations for switchgrass were scheduled based on heat units (Table 1). A base temperature of 12°C and an optimal temperature of 25°C were assumed [46]. The SWAT default value of 1426 total physiological heat units was assumed for switchgrass to reach maturity. As shown in Table 1, based on plot studies at various sites in Oklahoma, 85 kg ha^-1^ yr^-1^ of N fertilization was assumed for the simulation of switchgrass harvested as bioenergy feedstock [47]. At 120% heat units, the dry switchgrass yield was removed from the field using the “harvest only operation” of SWAT. The extra 20% of the heat units was added after maturity to dry the switchgrass stand in the field [30]. Every year of the simulation, winter wheat was planted on 30 September and harvested on 15 June, which are typical dates of planting and harvesting in Oklahoma. An auto-fertilization option with 0.9 stress factor triggering automatic fertilization was selected. No fertilization was assumed for the baseline grassland simulation because pastures and rangelands in the watershed are not typically fertilized.

**Table 1:** Management practices utilized in the SWAT model for the Skeleton Creek Watershed. Depending on the crop type either date or potential heat unit (PHU) was used when scheduling the operation.

Crop	Planting Date	Harvest Date	Fertilization date/ amount
Winter wheat	Sept. 30	June 15	Jan 15/ Auto fertilization initialization
Other Crops	0.15 PHU	1.20 PHU	0.16 PHU/ Auto fertilization initialization
Switchgrass	0.15 PHU	1.20 PHU	0.16 PHU/ 85 kg ha-1 of N
Other grasses (little bluestem)	0.15 PHU	1.20 PHU	No fertilization

### Streamflow calibration and validation

The SWAT model was calibrated for the monthly streamflow of the Skeleton Creek watershed from 2002 to 2007, and validated from 2008 to 2011 at the two gauge stations: Enid (upper gauge station) and Lovell (catchment outlet). These two gauge stations were used to better capture the effect of spatial variability of biophysical conditions on the streamflow during the calibration processes. The evaluation process consisted of three phases: sensitivity analysis, manual and auto-calibration, and validation. An automatic sensitivity analysis embedded in SWAT was used to select key parameters to be used for calibration. The sensitivity tool is based on a Latin Hypercube (LH) One-factor-At-a Time (OAT) sampling technique, where each run only has one parameter changed; hence it is clear that any change in the outputs is associated with that particular parameter [48]. To have a better understanding of the hydrological processes of the watershed and to make the calibration process more efficient, both manual and auto-calibration techniques were used. Using the most sensitive parameters, the model was first manually calibrated for the watershed by choosing parameter values that resulted in reasonable agreement between observed and simulated monthly flows for the two stations. After manual calibration, the SWAT2005 auto-calibration was employed using the sum of squared residuals (SSQ) as an objective function. During the calibration processes, first the streamflow at the upper gauge station was manually and automatically calibrated for the upper watershed. Then, the calibration result for sensitive parameters from the upper watershed was used as a starting number for manual calibration of the streamflow for the lower watershed. After manual calibration of the lower watershed, an auto-calibration was performed for its streamflow. After calibration was completed using the 2002–2007 data, the model was validated, at both stations, using the 2008–2011 data. The simulated monthly streamflow was compared with the observed streamflow at the two stations using three statistical tests: Nash-Sutcliffe Efficiency (NSE) [49], percentage bias (PBIAS), and coefficient of determination (r^2^).

### Land use change scenarios

The baseline simulation was created using the merged NLCD and CDL land cover data for the watershed. In addition to the baseline, two scenarios were developed based on our research objectives. Scenario-I simulates conversion of the NLCD’s “grassland/ herbaceous” land cover to switchgrass production. Around 376 km^2^ or 35% of the watershed was converted to switchgrass under scenario-I. Scenario-II simulates switchgrass production on class III and IV croplands. Around 289 km^2^ or 27% of the watershed was converted to switchgrass under scenario-II. Parameter values obtained during the calibration of the baseline simulation were also applied during scenario-I and scenario-II simulations to avoid artificial changes in the hydrological regime between scenarios. To compare the hydrologic impacts of these two conversion scenarios, we propose the “hydrologic cost-effectiveness ratio”, here defined at the HRU level as the reduction in the water yield (m^3^) from the HRU relative to the baseline simulation divided by the switchgrass biomass production (Mg) for the HRU. Cost-effectiveness ratios are often used in economic analyses to compare the relative merits of various courses of action, and cost-effectiveness ratios have been employed in some previous hydrologic studies [50, 51]. For example, Panagopoulos et al [52] used a cost-effectiveness ratio together with SWAT simulations to compare agricultural best management practices for a catchment in Greece.

## Results and discussion

### Streamflow calibration and validation

**Fig 3:**
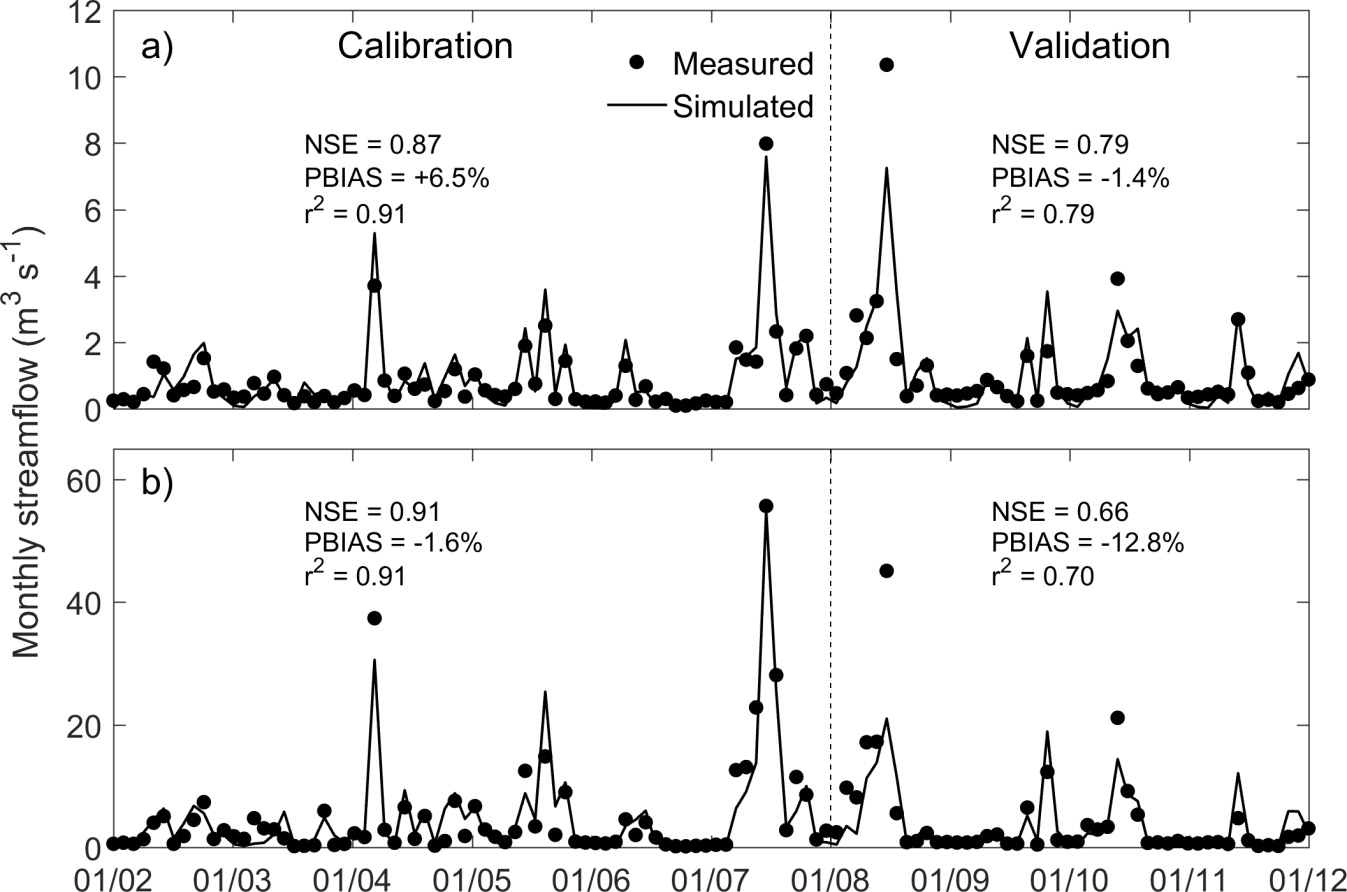
Comparison of observed and simulated monthly mean streamflow at Enid, OK (a) and Lovell, OK (b) during six years of calibration (2002–2007) and four years of validation (2008–2011).

Sensitivity rankings and final calibration values for key parameters are shown in Table 2. In the Skeleton Creek watershed, monthly streamflow predictions were most sensitive to curve number (Cn2). Soil evaporation compensation factor (Esco) was ranked second, and baseflow alpha factor (Alpha_Bf) was third in the sensitivity ranking. The NSE, PBIAS, and r^2^ for the calibration and validation periods at Enid and Lovell are shown in Fig 3. According to the performance ratings of Moriasi et al [53], model performance is good when NSE is greater than 0.65 and PBIAS < ± 15%, and very good when the NSE is > 0.75 and PBIAS < ±10%. By those standards, the performance of the calibrated model was good to very good. In addition to these statistical coefficients, from visual comparison it is clear that monthly simulated streamflow matched well with the observed streamflow at the two gauge stations during both calibration and validation (Fig 3). Thus, the SWAT model for the Skeleton Creek watershed was demonstrated to provide a reasonable hydrologic framework for testing our scenarios of changing grasslands to switchgrass (scenario-I) and marginal croplands to switchgrass (scenario-II).

**Table 2:** Sensitivity analysis and final calibration results for parameters influencing predicted streamflow for the upper basin (above Enid gauge station) and lower basin (between Enid and Lovell gauge stations).

Parameter Code	Description	Sensitivity ranking		Parameter changed to	
		Upper basin	Lower basin	Upper basin	Lower basin
Alpha_Bf^a^	Baseflow alpha factor (d^-1^)	3	3	0.1	0.18
Ch_K2^a^	Channel effective hydraulic conductivity (mm h^-1^)	9	5	5.62	3.12
Cn2^b^	Initial SCS CN II value	1	1	1.07	1.04
Esco^a^	Soil evaporation compensation factor	2	2	0.72	0.76
Gwqmn^a^	Threshold water depth in the shallow aquifer for flow (mm)	7	6	17.58	6.9
Revapmn^a^	Threshold water depth in the shallow aquifer for “revap” (mm)	10	7	33.04	55.35
Sol_Awc^b^	Available water capacity (mm H2O mm^-1^ soil)	5	4	1.018	1.085
Sol_Z^b^	Soil depth (mm)	6	8	1.028	1.021
Surlag^a^	Surface runoff lag time (d)	4	10	1.58	1.58

The parameter variation methods were a = replacement of initial parameter values with the new values, and b = multiplying the initial value by the calibration values

### Biomass production

**Fig 4:**
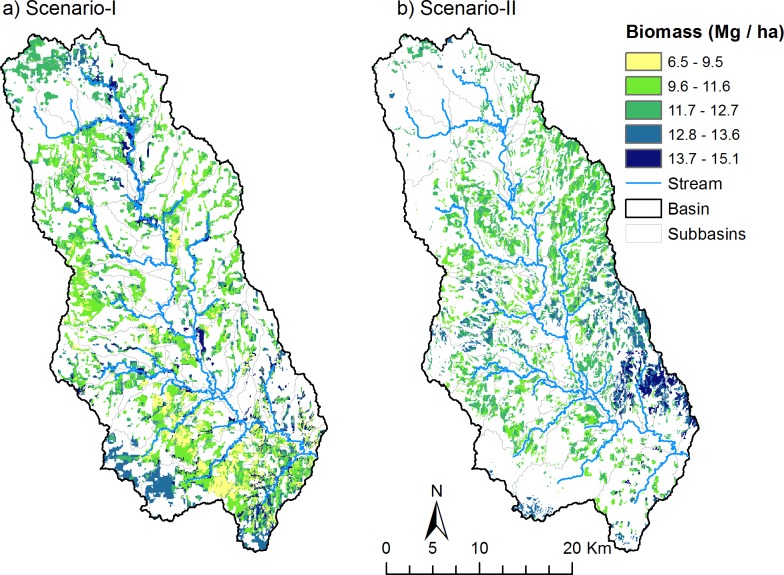
Simulated average annual switchgrass production for 2002–2011 for conversion of grasslands (a) and marginal croplands (b) to switchgrass.

For both scenarios, switchgrass biomass production was simulated for 10 years (2002–2011). On average, the annual switchgrass yield from the conversion of grasslands was 12.0 Mg ha^-1^. Biomass yield varied from 6.5 Mg ha^-1^ (usually around the crest of the sub-watersheds) to 15.1 Mg ha^-1^ on grasslands closer to the stream channels (Fig 4A). The average annual (2002–2011) switchgrass production on marginal croplands was also 12.0 Mg ha^-1^, varying from 8.2 Mg ha^-1^ in the north-central part of the watershed to 13.8 Mg ha^-1^ in the south-eastern part of the watershed (Fig 4B). For comparison, under the baseline simulation, the simulated average above ground biomass (both shoot and grain yield) for wheat on marginal croplands was 6.2 Mg ha^-1^ and the simulated average grass yield from existing grasslands was 1.8 Mg ha^-1^. The simulated biomass yield from the grassland is at the lower end of average annual values reported for hay production (excluding alfalfa) for the three counties in the watershed, which varies from 2 to 4 Mg ha^-1^ during the simulation period [3]. The simulated switchgrass yields were within the range of what has been observed in field trials at Chickasha, OK (13.5 Mg ha^-1^; [54]) and at Stillwater, OK (12.1 ± 4.5 Mg ha^-1^; [41]). In addition, our results agreed well with previous SWAT simulations of around 10 to 12 Mg ha^-1^ of switchgrass production for north central Oklahoma by Baskaran et al [30] in their simulation for the whole US. At 12 Mg ha^-1^ of switchgrass yield, the Skeleton Creek watershed would produce an average annual switchgrass biomass of 435,000 Mg if all existing grasslands were converted to switchgrass and 351,000 Mg if all marginal croplands were converted. These simulated biomass production levels are equal to or greater than the biomass feedstock needs of a cellulosic biofuel plant that was planned in this region. The Abengoa Bioenergy Biomass of Kansas plant near Hugoton, Kansas, USA had a goal to produce 25 million gallons of ethanol using around 350,000 Mg of biomass annually [55].

The 12.0 Mg ha^-1^ simulated aboveground biomass for switchgrass was dependent upon the specified 85 kg N ha^-1^ fertilization rate, which was previously found to be an optimal N rate for switchgrass in Oklahoma [47]. Changing this N rate for the SWAT simulation would affect simulated biomass production because there is sensitivity of biomass yield to N availability in the plant growth subroutine. For instance, simulated switchgrass biomass for the Skeleton Creek watershed varied from 3 to 14 Mg ha^-1^ as the N rate was varied from zero to non-limiting (“auto-fertilization” option in SWAT). Field experiments have shown that the actual sensitivity of switchgrass yield to N rate varies substantially with environment and initial soil fertility level [56]. Thus, some uncertainty in simulating the nitrogen response is unavoidable. Modelers should then be judicious about their choice of N rate in SWAT, and whenever possible SWAT switchgrass yield predictions should be compared to actual field measurements to ensure the simulation results are plausible.

### Hydrologic impacts

In the baseline simulation, annual water balance components averaged over HRUs for each land use type showed a lower ET and higher water yield from existing grasslands compared with marginal croplands (Table 3). On average, percentage of precipitation allocated for ET (i.e. ET/P) was 74% for grasslands and 88% for marginal croplands. Most of the remaining percentage of precipitation, 25% for grasslands and 11% for marginal croplands, was water yield (which includes surface runoff and base flow) to the stream (Table 3). Grasslands had steeper slopes (mean 2.8%) on average than the marginal croplands (mean 2.0%), which contributed to the greater predicted water yield from grasslands. In addition, grasslands had, on average, shallower soil depths (mean 1.3 m) than marginal croplands (mean 1.5 m). Likewise, the average water holding capacity (AWC) for soils under grasslands was 203 mm, whereas, AWC was 228 mm under marginal croplands resulting is a lower runoff under marginal croplands compared with grasslands. Simulated evapotranspiration from the existing grasslands was less than that from marginal croplands, in part because the unfertilized grasslands produced less biomass.

**Table 3:** Summary of average annual water balance (in mm) during the simulation period (2002 through 2011) for grasslands and marginal croplands in the baseline simulation, and switchgrass growing on existing grasslands and on marginal croplands along with the area occupied/converted (in ha).

Scenarios	Land use	Precipitation	ET	Surface flow	Base flow	Total streamflow	Area
Baseline	Grassland	814	599	148	55	205	37600
Baseline	Marginal Cropland	812	713	84	7	91	28900
Scenario I	Switchgrass	814	704	54	41	98	37600
Scenario II	Switchgrass	812	724	53	21	76	28900

Previous global studies showed that conversion of native grasslands to croplands reduced the ET and subsequently increased streamflow [57, 58]. Those findings might lead one to the erroneous conclusion that the existing grasslands in the watershed contribute less to streamflow generation than do the marginal croplands. Our results show that is not the case. In the baseline simulation, grasslands and marginal croplands occupy fundamentally different areas in the watershed, having different soil types and land surface characteristics and different management practices. The biomass production, ET, soil depth, and AWC for existing grasslands are less than those for marginal croplands, while the slope for existing grasslands is greater than that of marginal croplands. All these differences contribute to greater surface runoff from existing grasslands than from marginal croplands. Thus, in the Skeleton Creek watershed, about 50% of the streamflow was predicted to come from grasslands covering only 35% of the area of the watershed.

**Fig 5:**
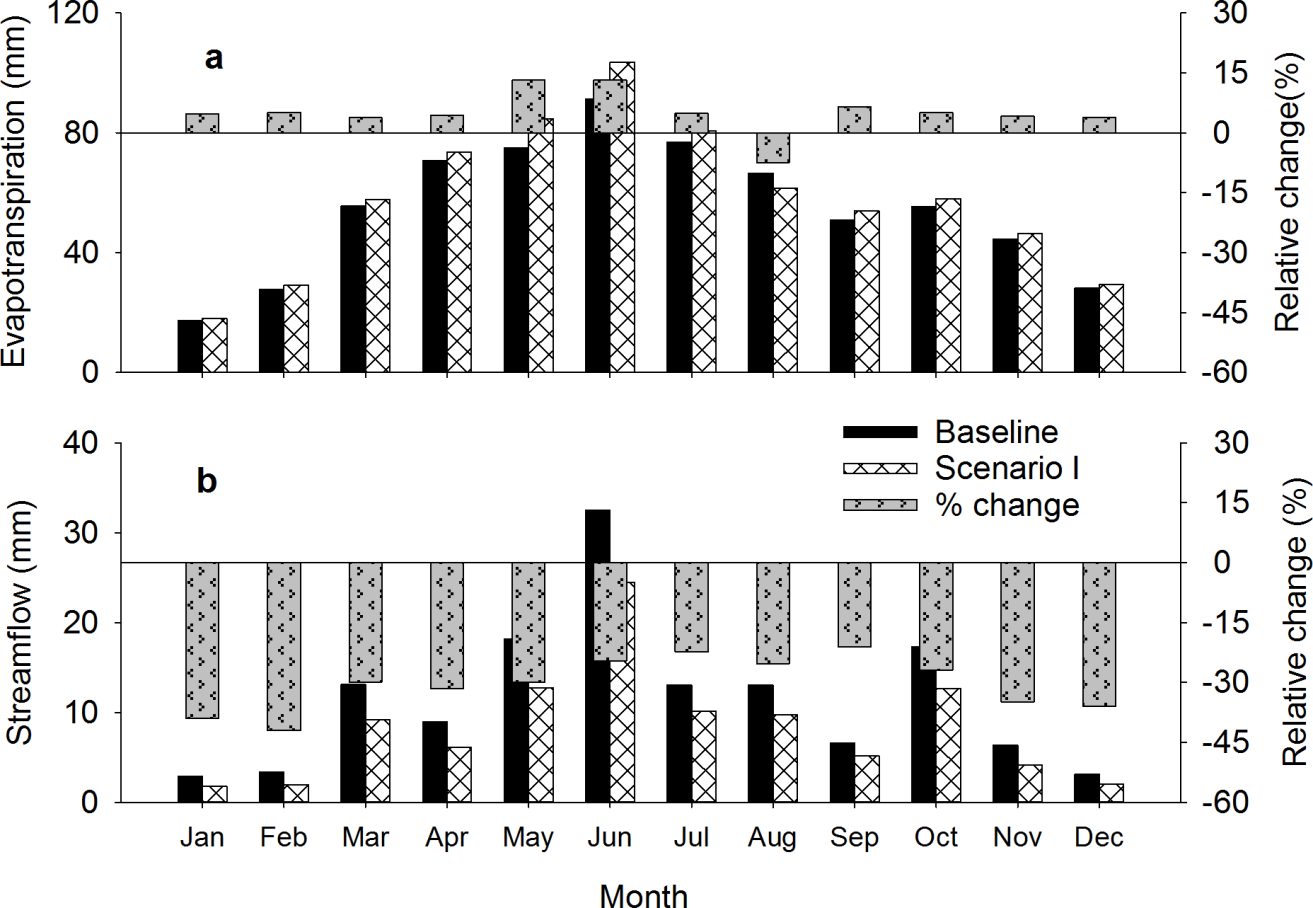
Simulated average monthly evapotranspiration (a) and streamflow (b) for 2002–2011 for the baseline and scenario-I (grassland conversion to switchgrass).

Conversion of existing grasslands to switchgrass (scenario-I) was predicted to increase ET in the Skeleton Creek watershed for every month except August (Fig 5A). For the baseline simulation, HRUs under grasslands had relatively low ET because they produced little biomass (1.8 Mg ha^-1^), but when grasslands were converted to switchgrass production with 85 kg N ha^-1^ fertilizer, the model predicted a 5.6% increase in annual ET under scenario-I compared to the baseline (Table 4). This increase in ET led to 21 to 39% reduction in average monthly simulated streamflow (Fig 5B).

**Table 4:** Summary of average annual water balance (in mm) for the entire watershed during the simulation period (2002 through 2011) for the baseline simulation, scenario-I (grassland conversion to switchgrass), and scenario-II (marginal cropland conversion to switchgrass) along with the area converted (in ha) and switchgrass produced (in Mg) under each scenario.

Scenarios	Precipitation	ET	Surface flow	Base flow	Total streamflow	Area converted	Biomass produced
Baseline	806	660	112	26	138		
Scenario I	806	697	79	21	100	37,600	435,000
Scenario II	806	663	104	30	134	28,900	351,000

**Fig 6:**
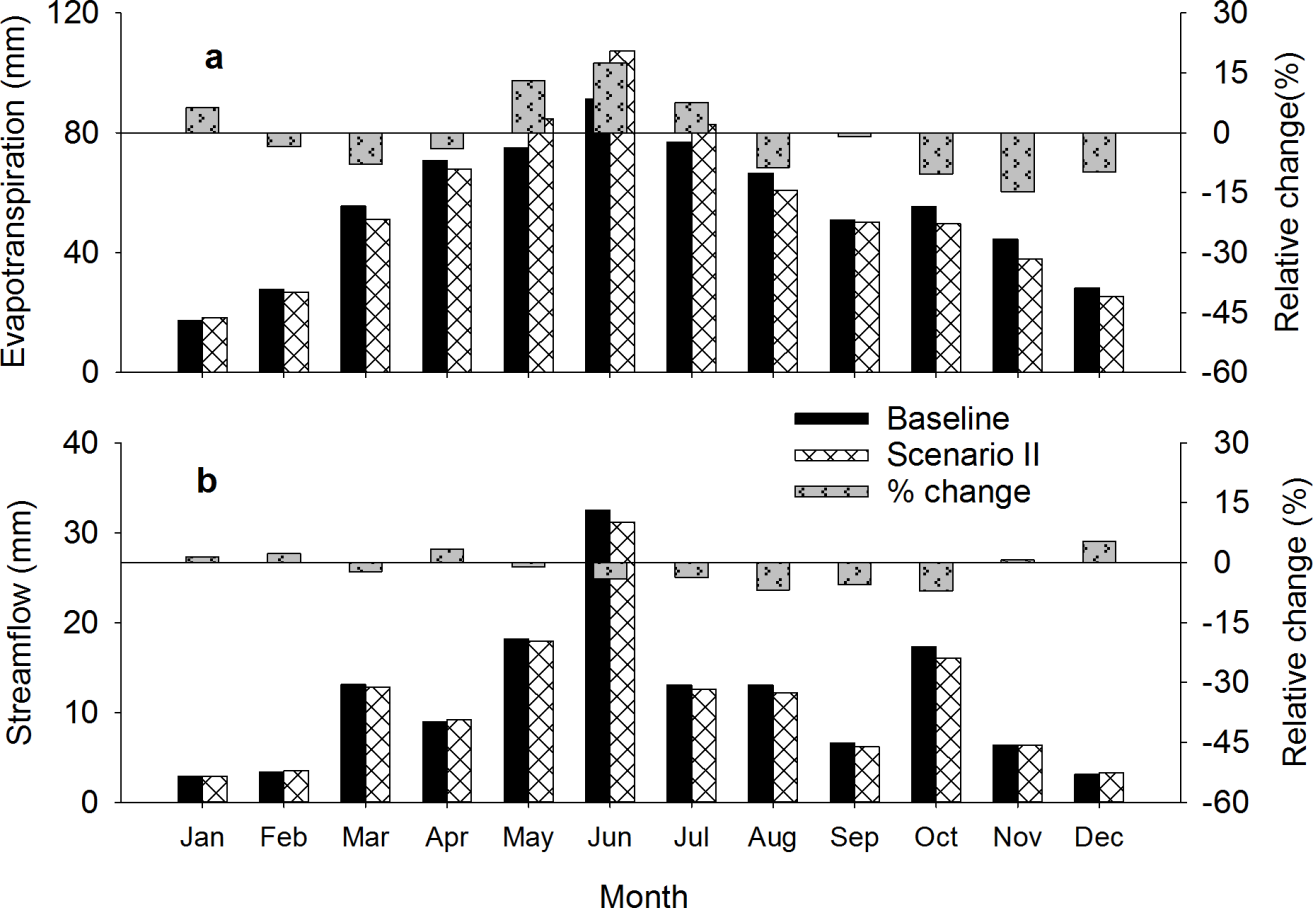
Simulated average monthly evapotranspiration (a) and streamflow (b) for 2002–2011 for the baseline and scenario-II (marginal cropland conversion to switchgrass).

In the baseline simulation, the dominant crop produced on marginal croplands was winter wheat. Changing this to warm-season vegetation (switchgrass) led to a partial shift in evapotranspiration from winter to summer (Fig 6A), although the difference in average annual (2002–2011) ET was small (Table 4). During May, June, and July, ET was up to 17% greater under scenario-II compared with the baseline because switchgrass was in its active growing period, and the winter wheat crop was under senescence or already harvested. The annual streamflow from the watershed was lower by 3.2% under scenario-II compared to the baseline (Table 4). The percentage reduction in monthly streamflow was greatest during late summer and early fall, but never exceeded 7% under scenario-II compared to the baseline (Fig 6B). For all the months, there was a simulated reduction in surface flow (0.01–2.16 million m^3^) and an increase in base flow (0.09–0.73 million m^3^), but the reduction in surface flow offset the increase in base flow, which yielded a reduced total streamflow (Table 4).

The simulated annual ET values for the major land uses (grasslands and cultivated crop lands) in this watershed agreed well with previously-reported measured ET values in the region. In our simulations, the average annual ET (2002–2011) for existing grasslands was 599 mm or 74% of annual precipitation. This is the HRU-level average ET considering only grassland HRUs, not the watershed-level average ET considering all HRUs. This simulated ET is consistent with measured annual ET for a native tallgrass prairie in north-central Oklahoma, which ranged from 637–807 mm or 53–73% of precipitation for the years 1997–1999 [59]. The somewhat higher annual ET and lower percent of precipitation used for ET in the Burba and Verma [59] study are expected because annual precipitation at their site was higher, ranging from 1104–1213 mm. The simulated average annual ET for crops (predominantly wheat) on marginal croplands was 717 mm, which was within the range of 714–750 mm of annual ET measured by Burba and Verma [59] for winter wheat in north-central Oklahoma. Likewise, the simulated average annual ET for switchgrass was 704 mm for switchgrass on grasslands (scenario-I) and 726 mm for switchgrass on marginal croplands (scenario-II), both of which are within the range of crop year ET values for switchgrass measured by Yimam et al [60] in Oklahoma. They found crop year ET values for switchgrass ranging from 627 mm at Stillwater, Oklahoma during a dry year to 846 mm at Chickasha, Oklahoma during a wet year.

Goldstein and Tarhule [13] also predicted reduced streamflow by 8.2% during winter, 26% during spring, and 44% during summer and increased ET by 44% during spring and 22% during summer in the Skeleton Creek watershed due to switchgrass production, but their conversion scenario involved 89% of the watershed area (both grassland and all cropland) being converted to switchgrass. It is not clear what driving factors would be necessary to result in such a dramatic land use change. In contrast, our scenarios involved conversion of 27–35% of the watershed to switchgrass and were predicted to produce adequate biomass to support a biorefinery. Wu and Liu [10] predicted a reduction in annual water yield to the stream by 2.1% by converting native grasslands (representing only 5.7% of the watershed area) to switchgrass production in the Iowa River basin. Clearly, at the watershed level, percent increases in ET and reduction in streamflow depend on the fraction of the watershed converted to switchgrass production, thus the conversion scenarios used in hydrologic studies should be critically evaluated.

The effect of converting cropland to switchgrass is, in part, dependent on the amount of biomass produced by the switchgrass relative to that produced by the displaced crops. For example, Wu and Liu [10] predicted an increase in annual average water yield of 1.7% when converting corn (*Zea mays*) croplands to switchgrass because biomass production from the corn was higher than that from switchgrass. But in the Skeleton Creek watershed the results are different, because the dominant crop, winter wheat, produced on average 6.2 Mg ha^-1^ above ground biomass on marginal cropland whereas the switchgrass was predicted to produce 12 Mg ha^-1^ on that same land. Converting the marginal cropland resulted in a significant shift in ET from fall and winter months to spring and summer months. This shift resulted in streamflow reductions June through October. These seasonal changes highlight the importance of considering shorter time scale variability of water balance components rather than looking only at the annual averages. This may be particularly important to maintain year-round “environmental flows”, which are the minimum streamflow levels required to achieve desired ecological objectives [61].

### Tradeoff between switchgrass production and water yield reduction

**Fig 7:**
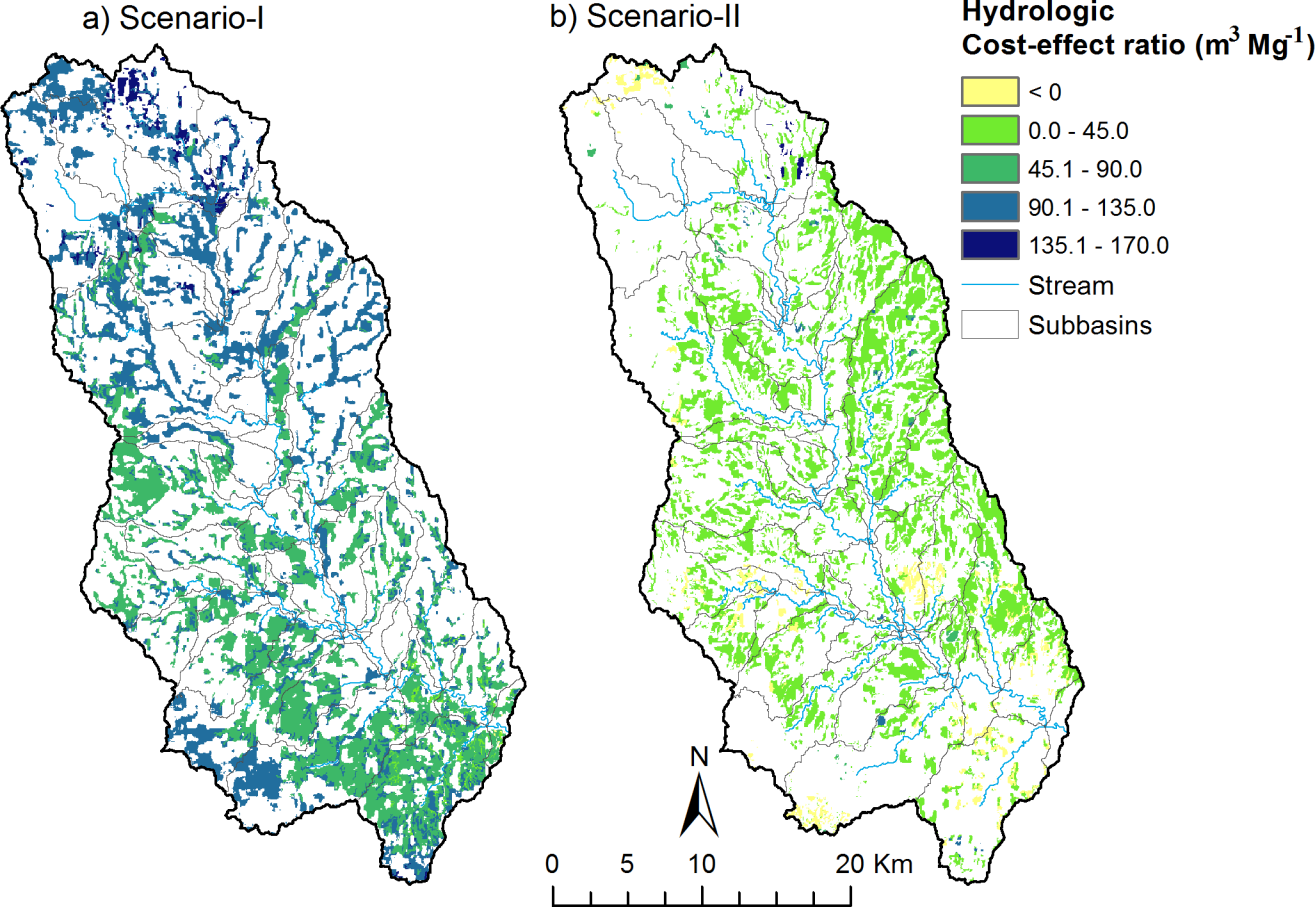
Map of hydrologic cost-effectiveness ratio for each HRU defined as average annual (2002–2011) water yield reduction relative to the baseline simulation divided by the switchgrass production for each HRU for the case of grassland conversion (a) and marginal cropland conversion (b).

Maps of the hydrologic cost-effectiveness ratio for scenarios I and II highlight the outcomes for grassland versus marginal cropland conversion (Fig 7). The water yield reduction (cost) per ton of biomass (effectiveness) for the grassland conversion scenario reached as high as 170 m^3^ Mg^-1^ in the northern portion of the watershed (Fig 7A) and was substantially higher than the cost-effectiveness ratio for marginal cropland conversion (Fig 7B). This higher cost-effectiveness ratio indicates a less desirable scenario. On average, in the Skeleton Creek watershed, to produce one ton of switchgrass on grasslands, the model predicted water yield reductions of 95 m^3^, while this value was only 17 m^3^ for the production of one ton of switchgrass on marginal croplands. Note that about 10% of the area in scenario-II has a negative cost-effectiveness ratio, which indicates an increase in water yield when marginal cropland is converted to switchgrass production on those HRUs. This increase was associated with differences in soil type and depth. The majority of soil types under marginal croplands have finer texture and deeper soil compared to those HRUs that have negative cost-effectiveness ratios, which tended to have medium textures and shallower soils. The higher hydrologic cost-effectiveness ratio for the case of grassland conversion, in this study, is consistent with the findings of Goldstein et al [12] who reported larger hydrologic impacts when converting grasslands versus winter wheat to switchgrass production in the SGP. However, their study did not normalize the hydrologic impacts by the amount of switchgrass produced to facilitate comparisons between scenarios.

## Summary and conclusions

The quest to produce cellulosic ethanol from plant biomass motivated us to investigate the interactions between bioenergy cropping systems and water resources. We used the SWAT model to evaluate the hydrologic effects and biomass production of two land use conversion scenarios for bioenergy cropping systems in the Skeleton Creek watershed in north-central Oklahoma. The simulations showed that, in this watershed, the average biomass produced following conversion of existing grasslands or marginal croplands to switchgrass was similar (12 Mg ha^-1^) but with greater spatial variability under the grassland conversion scenario. Under the baseline simulation, grasslands routed a greater proportion of precipitation to streamflow (25%) than did marginal croplands (11%). Conversion of marginal croplands representing about one quarter of the watershed area to switchgrass was predicted to reduce the annual streamflow by only 3.2%. In contrast, converting existing grasslands (35% of watershed area) to switchgrass production reduced simulated annual water yield to the streams by 27.7% and increased ET by 5.6%, in part because the fertilized switchgrass produced more biomass than the unfertilized existing grasslands. This study only addressed water quantity impacts, but conversion of land to switchgrass production could also have important implications for water quality, through changes in soil erosion and nutrient transport.

The hydrologic impacts predicted here are specific to the study watershed, suggesting the need for process based modeling to simulate outcomes in other specific watersheds. Policy makers may need to consider the tradeoffs between bioenergy feedstock production and reduction of streamflow and prioritize areas for bioenergy feedstock production accordingly. If the goal is to avoid streamflow reductions, planting switchgrass on marginal croplands may be preferable to converting existing grasslands to switchgrass. Marginal croplands are currently used for cultivation of grain crops, predominantly winter wheat in this area, even though these lands have severe to very severe limitations according to the LCC system. Conversion of these marginal croplands to bioenergy crops may raise controversial issues of land use for food versus fuel. However, even the grasslands are part of the food production system, as many are used for cattle grazing. If we pre-emptively eliminate marginal cropland from consideration for biofuel production, we may be increasing the probability of undesirable hydrologic impacts. Therefore, comprehensive assessments of bioenergy systems should include careful consideration of the impacts of land conversion on the hydrological regime.
